# Editorial: Credition—An interdisciplinary approach to the nature of beliefs and believing

**DOI:** 10.3389/fnbeh.2023.1217648

**Published:** 2023-05-23

**Authors:** Rüdiger J. Seitz, Hans-Ferdinand Angel, Raymond F. Paloutzian, Ann Taves

**Affiliations:** ^1^Department of Neurology, Medical Faculty, Centre of Neurology and Neuropsychiatry, LVR-Klinikum Düsseldorf, Heinrich-Heine-University Düsseldorf, Düsseldorf, Germany; ^2^Institute of Catechetics and Religious Education, Karl Franzens University Graz, Graz, Austria; ^3^Department of Psychology, Westmont College, Santa Barbara, CA, United States; ^4^Department of Religious Studies, University of California, Santa Barbara, Santa Barbara, CA, United States

**Keywords:** beliefs, social interaction, rituals, perception, religion, trust, appraisal, decision making

## Introduction

On the 20^th^ through the 22^nd^ of October 2021, the international symposium on “Creditions—An interdisciplinary Challenge” took place in the Conference Center of the Volkswagen Foundation in Schloss Herrenhausen, in Hannover, Germany. Due to the Sars2-Covid-19 pandemic the symposium had a hybrid format which allowed the participation of those unable to attend in person. Our aim from the outset was to publish a book based on the symposium presentations. Thus, we are delighted to introduce this e-book consisting of 42 chapters in total on the topic of belief and believing. We are grateful to the Volkswagen Foundation, Hannover, Germany, Frontiers Publishers, Lausanne, Switzerland, Siemens Healthineers, Erlangen, Germany, and the Anton-Betz-Foundation of the Rheinische Post e.V., Düsseldorf, Germany, for their generous support of this conference and publication of its proceedings.

The start of the credition project dates back to the first meeting under the auspices of the Karl Franzens University in Graz, Austria, in 2011 (https://credition.uni-graz.at/de/credition-research/). Our point of departure was the hypothesis that information processing in the human brain concerning external events in the environment and subjective internal states affords believing and predictive control of behavior (Seitz and Angel, 2012). Thus, this project focused on how to assess subjective experience with objective measures as discussed recently by Pauen and Haynes (2021). We hypothesized that the neural processes underlying believing constitute a domain similar to those for cognition and emotion, and therefore advanced the neologistic term “credition” to represent this domain (Angel et al., 2017). The plural form, creditions, is an umbrella term that signifies the neural subfunctions that constitute the category of processes of believing, as are similarly present in the categories of emotions, perceptions, and actions (Angel). The credition concept concurs well with the notion that internal states encode beliefs about the external world that involve belief formation, belief updating, and the transmission of beliefs to others, which yields shared beliefs (Albarracin and Pitliya).

The present chapter puts the contributions of this e-book into perspective in light of the current interdisciplinary research on belief and believing.

## Belief formation and updating

From the perspective of cognitive science, belief formation and updating result from the neuropsychological processes that afford believing. These processes consist of perception of external information, valuation in terms of subjective relevance or meaning, predictive coding of subsequent behavior, and encoding of this composite information in and retrieving it from memory (Seitz et al., 2018; Seitz and Angel, 2023). Perception involves unconscious multisensory integration within and across modalities and potentially conscious awareness of percepts that result (Firestone and Scholl, 2016). Because far more information is in the environment than any organism can perceive and process, organisms have evolved to perceive and process the information they need to survive. Humans and some other animals do so by segmenting information by a cognitive process that divides the information into chunks with a beginning and an end (Taves and Paloutzian). Furthermore, the processes of believing function to stabilize a given perception out of the myriad rapidly changing external stimuli in terms of personal meaning, which allows for socially adaptive behavior. Global meaning encompasses foundational beliefs, values and goals, and a subjective sense of meaningfulness, whereas situational meaning entails the appraisal of an experience (Park). Interestingly, the gut-brain-gut communication network is part of the interoceptive circuits that enable a person to sense and interpret the physiological condition of the body and regulate its autonomic and mental activity (Holzer). Ultimately, the processes of believing constrain an individual's behavior in a stochastically predictable way (Seitz et al., 2018). As humans trust their beliefs, the beliefs provide a temporarily reliable link between a person's past experience and his/her future behavior.

With respect to the bottom-up processing of external information that can become the object of believing, three categories of beliefs have been differentiated: (a) empirical beliefs about objects and facts, (b) relational beliefs about events including human interactions, and (c) conceptual beliefs about narratives including those held in societies (Seitz and Angel, 2020). Empirical and relational beliefs occur below the level of awareness, and thus correspond to so-called primal beliefs as depicted in a multi-level scheme (Table 1). In contrast, conceptual beliefs are mediated by language and are objects of conscious awareness. This conceptualization accounts for the hierarchically nested structure of the three-levels of believing processes, i.e., the physical level, the interpersonal level, and the social level (Sugiura et al., 2015). In essence, the ability to believe expands human cognitive, sensory, and perceptual dynamics and is essential for the human ability to engage with and shape the world, as is evident from phylogenetic evolution (Fuentes). This accords with van Eyghen' claim that explaining belief and believing from an ontogenetic perspective is more parsimonious than from a phylogenetic account, because there is no need to postulate anything beyond the plasticity of the human brain and mind.

**Table 1:** Beliefs as the result of the processes of believing.

**Input level**	**Objects**	**Events**	**Narratives**
First person level	I believe that	I believe him/her	I believe in
Third person level	Empirical beliefs	Relational beliefs	Conceptual beliefs
Meta analytic level	Primal beliefs		Autobiographic Religious Political

For comparison, placebo effects rely on the brain's ability to integrate contextual information in the environment with prior experiences, and are likely due to emotional re-appraisal strategies and cognitive-evaluative processes. Only very strong placebo interventions, such as those induced by classical conditioning, may affect early sensory processes in a significant manner (Meissner). Upon believing, we assume that what we believe is true and that it correctly reflects the environment. Thus, what we personally believe is true may be mistaken, e.g., by visual plausibility (Adelson, 1993) or by confused timing of thought (Bear et al., 2017). For reasons of space, we do not enter the discussion of the different concepts of truth (McLeod, 2021) nor do we refer to different functions of truth, as for instance in the debate about “narratives” (Mercier, 2020). Rather, we refer to the empirical evidence that truth judgments are based on a bias to judge incoming information from the environment as true, so long as there is an ease of processing whether assertions match information stored in memory (Brashier and Marsh, 2020). Sensory perceptions are typically processed with ease and thereby construct our experience of our environment. This is no different from how language-based information is assembled. Typically, repetition of statements that facilitate the subjective ease of processing has been shown to increase the likelihood that a statement is judged true (Wang et al., 2016). Nevertheless, language comprehension can be difficult and may even require a third person's interpretation. Usually, what a person says or does is taken to reflect what he/she is believing. In fact, belief congruity, social congruence, and message repetitions have been proposed to enhance the probability that implausible and false information may be accepted as true (Levine, 2022). However, deceptive intentions by other persons have to be taken into consideration as well. Accounting for these different aspects, Connors and Halligan have proposed a five-stage model for belief formation that involves a triggering sensory precursor, meaning attribution, belief evaluation, belief acceptance, and effects of beliefs.

In accordance with these observations we claim that humans, like non-human primates, are engaged in believing that their senses provide a true image of their environment. Although there are reasons to decide amongst alternatives about how to behave, to believe is a mandatory function which enables a subject to develop preferences to regulate behavior in an ecologically adequate fashion in a complex environment. However, there are non-evidential reasons, be they embedded in religions, worldviews, or secular ideologies, for believing (Longheed and Simpson, 2017). Similarly, the notion that some of our beliefs are under our control—we manage the cognitive mechanisms that issue them and control whether they operate in the right environment (Visala)—makes it likely that we will underestimate the fluidity of beliefs brought about by new information from the environment (Seitz et al., 2018). This fluidity needs to be differentiated from the colloquial saying, “There is good reason to believe that...,” because it is a meta-cognitive statement from a third person perspective. Such a statement conveys that the person who is stating it judges the thing in question to be similar to how it is judged by the person whose behavior he/she observed.

## Neural processes underlying believing

The neural representations involved in the formation and updating of primal beliefs about objects and beliefs are pre-linguistic in nature and are maintained in large-scale cortico-subcortical networks in the human brain (Seitz). The cortical structures involved include the dorsolateral prefrontal cortex, the parietal cortex, and the so-called pre-supplementary motor area in the dorsomedial frontal cortex. When people believe that they have recognized a target, this network including subcortical structures like the basal ganglia, thalamus, and amygdala become active as was shown in a functional magnetic resonance imaging (fMRI) study when subjects were asked to indicate when they recognized emotions in slowly evolving facial stimuli (Sonnberger et al.). Of particular relevance are the brain structures that are part of the affect regulating system. For example, in another fMRI study it was found that the belief that a leader is transformational triggers neural activations in the follower's reward circuitry that correlate with the follower's level of motivation (Bergner et al.). Most recently, a large fMRI study on more than 900 volunteers has shown that emotions can enhance memory encoding of pictures which is mediated by a large circuit of interconnected brain areas including cortical areas, the hippocampal formation, the amygdala as well as the thalamus and cerebellum (Fastenrath et al., 2022). Moreover, when subjects were required to listen to stories, fMRI revealed an enhanced activity in the widespread cortical semantic system related to specific semantic domains or groups of related concepts (Huth et al., 2016). Conversely, transient inactivation of these areas, the left inferior frontal gyrus, by transcranial magnetic stimulation was found to reverse the habitual tendency to discount bad news in belief formation (Sharot et al., 2012).

A meta-analytic research project revealed that mindfulness can be acquired by meditation techniques and lead to emotional regulation, and to monitoring perception and behavior with particular emphasis on increasing the experiential phenomenological self and reducing self-relational thoughts of the narrative self (Weder). Self-referential thinking during mindfulness and self-relational thinking in the narrative self relies on the default mode network including the dorsal and medial prefrontal cortex, and posterior cingulate cortex. These findings correspond well to the notion that self-estimates of abilities like self-esteem, self-concept, and self-efficacy are conceptually close to beliefs (Neubauer and Hofer). Furthermore, it has been suggested that the common cognitive bias underlying the multidimensionality of self-transcendence is related to a sense of self-agency, indicating the possibility that the bias is caused by a process that controls the neural networks involved in multilevel forward model prediction (Sugiura). From a phylogenetic point of view it is noteworthy that when monkeys viewed other monkeys, a number of processes took place. They included the recall of novelty and emotional significance from memory of previous experiences with other macaques, the novelty of the individual seen in a mirror, innate fear, etc. (Bretas et al.). Specifically, the belief that the macaque in the mirror is a reflection of the self was found to be expressed in the form of mirror self-recognition behavior.

Social interactions of individuals rely on believing the bodily and verbal expressions of the counterpart, which can be suspected to involve empathy. Using a new model of empathic learning using a feedback loop it was found that changes in inter-brain coupling in the inferior frontal gyrus represent a core component of affect empathic reactions (Shamay-Tsoory). Moreover, an embodied approach to abstract words and cognitive concepts may shed light onto the process of building and revising beliefs, specifically suggesting that beliefs, much like other conceptual domains, can be grounded in actual experiences and their complexity (Buccino and Colagè). Furthermore, brain imaging results in healthy volunteers of Caucasian and Chinese ethnicity suggest that the development of culturally specific beliefs is brought about by culture–brain interactions via the practice of behaviors and by direct culture–brain interactions that are based on distinctive neurocognitive processes (Han et al.).

## Beliefs as conceptual expressions

Only a small proportion of information enters someone's conscious awareness and can then be expressed from first-person perspective as “I believe …” (Oakley and Halligan, 2017; Seitz and Angel, 2020). Such a proposition is a probability statement that signifies by means of verbal behavior an affective involvement of the speaker. It is used with a slightly different phrasing for objects, events and narratives as summarized in Table 1. These statements are different from a confidence statement (Ülkümen et al., 2016). Accordingly, people use the verb “believe” in a highly differentiated fashion and in different contexts compared to how they use the verb “think.” Empirical evidence suggests that people use “believe” preferentially in religious contexts, whereas they say “think” when they refer to a confidence statement about facts (Heiphetz et al., 2021). In contrast, it is uncommon to use the noun belief for such a statement (It is my belief that …), although it is a common expression from the third-person perspective (It is his/her belief that …). Typically, the content of such a belief is specified in certain areas of discourse such as religion, morality, politics, etc. Although commonly done in English, one should be aware that labeling is a post-hoc attribution from a meta-analytic perspective (Seitz et al., 2022). Thus, the belief in question is brought about by inferential thinking of an observer and attributed to a behavioral outcome such as a verbal statement or an action. Accordingly, the labels *political, religious, moral*, and *social* belief involve the tacit claim that believing can be classified from a third-person top-down perspective according to putative epistemological entities, such as religion, politics etc., (Table 1). In fact, these entities are language-based narratives that represent what we have called conceptual beliefs (Seitz and Angel, 2020). Probably related to a teleological view, the specificity of such conceptual contents of beliefs has been questioned (Oviedo and Szocik, 2020). Furthermore, a *post-hoc* attribution is hardly compatible with a general neuroscientifically grounded model of belief formation and updating, as realized in parallel organized cortico-subcortical networks affording predictive processing (Friston et al., 2017; Seitz et al., 2018).

It is important to realize that there are intriguing linguistic issues concerning the notion of beliefs and believing (Angel). In English one can speak about beliefs in plural. In contrast, in German the term for belief appears monolithic, as it does not have a plural form. When one has to translate beliefs (plural) into German, most likely instead of belief (=GLAUBEN) another term (MEINUNG = opinion) will be used because it can occur in both the singular and the plural form. Yet, one has to acknowledge that “opinion” lacks an affective meaning, in contrast to “belief.” Thus, texts that were translated from English into German may suffer the lack of linguistic clarity. For instance, one can have a religious GLAUBEN but religious MEINUNG does not make sense. Furthermore, in German there is no equivalent term for believing. In contrast, the phrase “processes of believing” can be translated into German. Therefore, we have to acknowledge that how language is used indicates cognitive assumptions about prior knowledge that is likely to influence the adoption of new information and conclusions (Madzarevic). Nevertheless, credition was said to afford openness of the self to the freedom and play that are fundamental to being human (Davies).

Notably, there is a tight link between belief and knowledge, as knowledge has traditionally been defined in philosophy as justified true belief. It is important to note, however, that Popper replaced the problem of justification with the issue of criticism, which is an argument for a fluid character of beliefs (Diller, 2006). Nevertheless, from a philosophical point of view beliefs have been dichotomized into categorical (yes/no) beliefs and graded beliefs. While the former are logically coherent and deductively closed, the latter are probabilistically coherent with a probability of < 1.0 (Dietrich). Likewise, doxastic logics lead to propositions concerning beliefs (“it is believed that”), whereas deontic logics result in prescriptions (“it is obligatory that”). The interesting question is how these beliefs can be revised Vestrucci). Beliefs, however, may also reflect a property of the believing person. For example, according to the concept of representationalism, a given representation with the content P may be deployed in reasoning. For comparison, according to dispositionalism, a person may believe a given proposition, because she/he is disposed to act and react in this way (Schwitzgebel). Furthermore, the belief that a person is epistemically confident about something is likely to be formed and revised differently from a belief that is central to a person's identity or heart (van Leeuwen). Nevertheless, one should be aware that these discussions deal with *post-hoc* theoretical reasoning but not with cognitive science of belief formation and updating.

## Abnormalities of believing

Diseases of the brain may disrupt any of the processes of belief formation and updating, as for example in the alien limb syndrome, agnosia, hallucinations, and delusions (Seitz, 2022). For example, empirical studies have shown that in altered sensorimotor processing, self-monitoring can link hallucinations of presences to the detection of human agents (Vehar et al.). From a pathophysiological perspective it is noteworthy that brain lesions affecting the dorsolateral and ventromedial prefrontal cortex as well as the posterior superior temporal cortex were found to facilitate the occurrence of religious beliefs, mystical experience, and ideological commitments (Cristofori et al.).

Furthermore, after traumatic experiences people have been shown to make meaning to reduce discrepancies between situational and global meanings, with a greater reduction in the size of discrepancies predicting better adjustment following trauma (Park). Similarly, in the Covid-19 pandemic, patients with affective disorder were more uncertain and experienced fewer positive emotions than healthy controls, although both groups did not differ in vaccination status (Dalkner et al.). Particularly, in psychotic disorders and a wide range of other neuropsychiatric conditions abnormalities of belief formation may result in discrepancies between bodily expressions and verbal reports. Such discrepancies may cause distrust in the addressee(s) and eventually may destroy social bonds. However, because beliefs are subject to change, people may adapt their behavior and can create new experiences—often during social interactions—which may help them to leave abnormal beliefs behind (Pott and Schilbach) and facilitate the speculation that psychotherapeutic interventions may become operative via socio-verbal interaction.

## Believing enables decisions

Decision making has been the object of scientific research for many years opening broad perspectives in the theoretical and practical areas of the sciences. The neural processes affording decision making have been studied in animals including non-human primates and mammals as well as in humans using neurophysiological and neuroimaging techniques. Moreover, the roles of attention, perception and choice-consistency have been explored recently (Nitsch and Kalenscher, 2021). Owing to the notion that meaning making and affective relevance are inherent in the processes of belief formation, a tight link between believing and the establishment of preferences can be postulated. Preferences allow for predictive coding and, thus, are key factors in decision making and selection of behavior. Empirical findings support the notion that our preferences evolve endogenously during the process of making decisions between equally preferred items (Voigt et al.). Therefore, self-determined, subjective cognitive concepts, such as our preferences, might be emergent consequences of the particulars of the decision scenario itself. Findings from functional neuroimaging studies support the view that the orbitofrontal cortex contributes to expectation-guided decision-making by enabling us to simulate the consequences of our choices (Kahnt). Moreover, it was found in choice tasks that value of the items and confidence in the decision involve large parts of the medial prefrontal cortex with a specific activation for value in the ventral portion and for confidence more dorsally in the anterior portion (Claris and Pessiglione, 2022).

Against this background and with respect to many failed engineering projects, it ought to be questioned whether engineers usually make rational decisions during product development. How to support decision-making is therefore a central topic in complex decision situations (Kranabitl and Faustmann) including economic decisions. For such purposes, an elaboration of artificial intelligence modeling of the capacity to reflect, rationalize, and communicate has been developed to support and even improve decision making (Lumbreras).

## Believing and social life

Individuals are members of social groups. After birth these groups are families that used to belong to tribes and, nowadays, typically are inhabitants of a village or town. Given these contexts, a person's behavioral decisions can be expected to evoke reactions in those to whom they are addressed or in other group members who are bystanders. To review the wealth of historical, philosophical, anthropological and psychological literature on this issue would far exceed the limits of this article. Nevertheless, in what follows we highlight some aspects of creditions that are pertinent to the relationships between believing and social life.

It is well-known that people can communicate the content of their beliefs as personal statements and can repeat the statements of others to themselves or other people. The power of language is that we can express our thoughts and emotions verbally, although we need to accept that in describing emotions and thoughts we are limited by the words we use (Abukhalaf, 2021). Linguistic research has shown that beliefs are based on the reliability and solidity of our knowledge and are typically described by abstract rather than concrete concepts (Borghi et al.). Thus, verbal expressions enable us to begin to understand the conceptual beliefs of other people. Above that, the exchange of verbal information typically benefits from the consistency between a person's verbal statements and his/her bodily expressions, because the person then appears particularly trustworthy (Seitz). Importantly, the transmission of narratives among members of a group can lay the groundwork for social cooperation within and possibly between groups. Whereas reputation has been found to sustain cooperative relationships among unrelated individuals in social groups and systems (Romano et al., 2021), another key to promoting prosocial behavior within a group may be reciprocation among the group members (Teehan, 2006). None the less, morality comes into play here because it promotes within-group cohesiveness and empowers individuals to protect their offspring (Teehan, 2006). Interestingly, the expectation to behave morally is not necessarily extended to individuals outside one's own group.

Consequently, it has been proposed that believing includes a component of trust that can be expressed in verbal communications, including those that convey information beyond one's personal experience. This degree of acceptance or trust probably also applies to news as well as to norms and promises within social groups. Granting trust may thereby be considered as a basis for social cooperation and group cohesion. It has been shown that the assignment of trust is learned by employing predictive coding, as is manifest in the processes of believing (Seitz). At the neural level, learning about the assignment of trust has been shown to involve the medial frontal cortex for confirmatory evidence of trust and to involve the lateral prefrontal cortex for alternative, untrustworthy outcomes (Akaishi et al., 2016). Thus the processes of believing are important neural functions that may ultimately be the springboard for the evolution of human social life and the development of culture and civilization (Fuentes). Thus, although believing is essential for creating preferences that afford behavioral decision making (see above), these processes are continuously modified by confirmatory or contradictory information (see above). Accordingly, moral and social beliefs are not stable entities that change only when there is dissonance between them (Dalege and van der Does, 2022). This view casts doubt on the notion that moral beliefs are necessarily explicit conceptual *post-hoc* descriptions (see above) and that beliefs about social networks are by definition implicit and formed in a pre-linguistic fashion (Korman et al., 2015; Seitz and Angel, 2020). Consequently, these different types of beliefs have to be assumed to compete within conscious and unconscious awareness. This raises the interesting question of whether the stability (or changeability) of beliefs is due more to external information or the individual's affect, particularly in different times or contexts.

## Believing and religion

In recent years, there has been an increasing interest in the multilevel interdisciplinary research on the cultural evolution of religion and spirituality (Paloutzian and Park, 2013; Feierman and Oviedo, 2020). Originally, the Ancient Greek terms for *belief* and *to believe*, namely πíσ*τις* *(p*í*stis)* and π*ιστε*úε*ιν* *(pistéuein)*, were not exclusively related to religious experiences. But from about the 4^th^ century onwards the expansion of Christianity linked beliefs more and more with *Christian beliefs*. Thereafter, since the end of the Middle Ages, the notion *religious beliefs* became common. In consequence, the term and the concept(s) of *religion* became predominant as a conceptual framework for understanding religious experiences. Regardless that the understanding of religion changed profoundly during history, the development of the concept of religiosity was virtually neglected (Angel, 2022**)**. From the cognitive science perspective, however, religiosity, not religion, is the relevant focus for understanding religious experiences (Angel, 2020). More recently, the evolutionary and cognitive accounts of religious beliefs have challenged the justification for believing in religious propositions (Teehan, 2014). Justified religious beliefs have been defined as beliefs that are consistent with the beliefs and grounds of belief employed in a given belief tradition (Teehan, 2014). It is widely accepted that religious beliefs exert a profound social impact. On the individual level they have the pragmatic aspect that they allow persons to make sense of their lives and of the world they live in. On the social level, they are said to promote inter-individual cooperation and to regulate inter-group conflict and competition within ethnic groups (Norenzayan et al., 2016). This is probably enhanced by ritualistic synchrony in religious acts that has been found to play a key role in cultural evolution (Gelfand et al., 2020). In correspondence with this notion, Geertz (2013) proposed that the co-evolution of genes and culture is a mover of the cultural evolution of religion.

It has been suggested that religious beliefs are brought about by a number of deeply engrained psychic functions such as agency detection, mentalizing, or dualistic reasoning (van Elk). People seemingly tend to attribute significance to information from sources they deem trustworthy. Specifically, empirical evidence points toward the role of cultural scaffolding and explicit teaching for endorsing supernatural beliefs (van Elk). Furthermore, in empirical studies on over 2,000 participants from different religious traditions in the United States, Ghana, Thailand, Vanuata and China, it was found that the power of the cultures in combination with individual differences shapes what feels real to the senses such as gods and spirits (Luhrman et al., 2021). Also, based on the exploration of classical Buddhist theories, Jed Forman argues that higher-order cognitive processes, like reflection on beliefs, may not only manipulate how we see our environment but also may generate a platform for what we see. Consistent with this notion, it has been proposed that various ways to purify the mind and develop its potential can be found in ancient Buddhist sutras (Du). It is noteworthy that Islamic thought contains opinions, positions, and sayings that have been transcended in many respects to keep pace with the current questions and developments posed by the socio-cultural environment of contemporary Muslims. Therefore, the Editors regret that of the two papers addressing the Islamic background one was withdrawn by the authors while the other did not pass the publication process. Even so, we hypothesize that the fundamental logic that underpins the processes of believing applies not only to everyday life, but also to religions including Islam.

Conversely, in Western countries the increasing number of non-religious people are moving away from the traditional religious narratives that provided meaning and structure around the dead body for both themselves and others (Applewhite). Consequently, they also introduce new kinds of meaning that are likely to affect values and beliefs around environmentalism, secularism, economics as well as traditions outside of religion. Observations of this sort may raise questions about the decreasing appeal of the promises that are central in traditional religious belief systems but similarly also in political ideologies.

## Conclusions

This e-book provides an up-to-date overview of how the introspective experience of believing something can be an issue of cognitive science and philosophy (Pauen and Haynes, 2021). On the behavioral or phenomenological level we have summarized the accumulating evidence suggesting that believing involves bottom-up processes that empower humans to select their behavior according to implicit as well as explicit, e.g., verbally coded, preferences. Also, we have described that the resulting beliefs typically are labeled semantically from a *post-hoc*, third-person perspective based on top-down inferential thinking. On the neural level the processes of believing were shown to be implemented in large-scale brain circuits. Whether functional imaging can show neural processes or representations such as social event knowledge or beliefs is an issue of a long discussion (Krueger et al., 2009). This e-book does not pertain only to the biological sciences but also to the theoretical sciences and the humanities. We hope that it can stimulate empirical and theoretical work to elucidate the driving forces of how humans have shaped their civilizations as well as the foundations of art.

## Author contributions

RJS: writing. H-FA, RFP, and AT: editing. All authors contributed to the article and approved the submitted version.
